# Atrial fibrillation is associated with increased in-hospitality mortality during Chimeric Antigen Receptor T-cell therapy hospitalizations: a retrospective cohort study in the United States

**DOI:** 10.1186/s40959-025-00334-5

**Published:** 2025-07-03

**Authors:** Nischit Baral, Nabin R. Karki, Daniel A. Ladin, Raja Zaghlol, Mahmoud Ibrahim, Alexander Rabadi, Tarec K. Elajami, Olivia Mechanic, Arvind Kunadi, Joshua D. Mitchell

**Affiliations:** 1 Cardiovascular Division, Mount Sinai Medical Center, Columbia University, Miami Beach, FL USA; 2 Department of Internal Medicine, Mclaren Flint/Michigan State University College of Human Medicine, Flint, MI USA; 3 Mitchell Cancer Institute, University of South Alabama, Mobile, AL USA; 4 Cardiovascular Division, Department of Medicine, Washington University School of Medicine, Campus Box 8086, 660 S. Euclid Avenue, St. Louis, MO USA

**Keywords:** Chimeric Antigen Receptor T-cell therapy, Atrial fibrillation, Cohort Study, In-hospital Mortality, Cardiovascular disease, Cancer

## Abstract

**Background:**

Chimeric Antigen Receptor (CAR) T-cell therapy (CAR-T) has emerged as a promising treatment for specific hematological malignancies. While some studies suggest an association between CAR-T and atrial fibrillation (AF), more data are needed on the association of AF with CAR-T outcomes.

**Methods:**

This retrospective cohort study utilized the National Inpatient Sample (NIS) 2017–2020 to explore in-hospital outcomes in cancer patients with AF while undergoing CAR-T. Comparisons were drawn between patients with and without AF during the hospitalization, assessing various parameters including mortality rates, length of hospital stay, and occurrences of acute heart failure, pulmonary edema, and gastrointestinal (GI) bleeding.

**Results:**

Of the 236,270 cancer-related hospitalizations, 1,030 cases (0.44%) received CAR-T. The average age of CAR-T recipients was 55.6 years ± 18.1 years, and females constituted 40.5% of the total CAR-T recipients. Of the 1030 patients receiving CAR-T, 97 (9.4%) had an associated diagnosis of AF during their hospitalization. A multivariable logistic regression analysis, adjusted for age, sex, race, comorbidity, and income, revealed that hospitalized cancer patients who underwent CAR-T therapy with AF had increased odds of in-hospital mortality (adjusted odds ratio, aOR: 3.87), acute pulmonary edema (aOR: 3.29), GI bleeding (aOR: 5.46), acute heart failure (aOR: 10.2), and extended hospital stays (Beta coefficient: 0.18) compared to hospitalizations with CAR-T but without AF. Similar results were observed in two sensitivity analyses: one limited to patients with diffuse B-cell lymphoma, and another excluding patients who had sepsis or respiratory failure while receiving CAR-T therapy.

**Conclusions:**

In cancer patients receiving CAR-T, inpatient AF is independently associated with a higher risk of in-hospital mortality, acute pulmonary edema, gastrointestinal bleeding, acute heart failure, and prolonged hospitalization.

**Supplementary Information:**

The online version contains supplementary material available at 10.1186/s40959-025-00334-5.

## Highlights

1. Hypotension and arrhythmias are common adverse cardiovascular events in hospitalized patients receiving Chimeric Antigen Receptor T-cell therapy (CAR-T).
2. Atrial fibrillation was present in close to 10% of patients receiving CAR-T while hospitalized.
3. Atrial fibrillation (at any time) was associated with increased in-hospital mortality, gastrointestinal bleeding, acute heart failure, pulmonary edema, and extended hospitalizations in cancer patients receiving CAR-T.

**Supplementary Information:**

The online version contains supplementary material available at 10.1186/s40959-025-00334-5.

## Background

Over the last decade, Chimeric Antigen Receptor (CAR) T-cell therapy (CAR-T) has emerged as a revolutionary tool for cancer treatment. In its original design, a CAR was composed of an antibody-derived single-chain variable fragment (scFv) fused to the T-cell receptor (TCR) signaling domain, intended to redirect T-cells to target solid tumors and human deficiency virus (HIV) [1, 2]. With time, the design of CAR T-cells evolved to include costimulatory domains and improved ex vivo culturing methodologies [1]. In 2017, the Food and Drug Administration (FDA) approved tisagenlecleucel and axicabtagene ciloleucel for the treatment of relapsing/refractory diffuse B-ALL (B-acute lymphoblastic leukemia) and diffuse large B-cell lymphoma (DLBCL).

Despite its therapeutic promise, CAR-T may have potential life-threatening adverse events, including cytokine release syndrome (CRS) and immune effector cell-associated neurotoxicity syndrome (ICANS). In addition, cardiotoxicity affects up to 26% of cancer patients who undergo CAR-T, [3] and may share pathophysiological pathways overlapping with CRS and ICANS. CRS is associated with various cardiovascular manifestations, including arrhythmias, heart failure, cardiogenic shock, and cardiomyopathies [4]. In a recent retrospective study, atrial fibrillation (AF) was the most common arrhythmia in CAR-T recipients [5]. However, there are limited data on whether AF is associated with higher mortality and other adverse clinical outcomes in CAR-T recipients hospitalized in the US. Our study aims to investigate the association between CAR-T and AF in the National Inpatient Sample (NIS), including potential correlations between AF and in-hospital outcomes in this cancer population.

## Methods

This retrospective, population-based cohort observational study drew from all hospitalizations in the NIS from January 1, 2017, to December 31, 2020. NIS is the largest inpatient database in the United States and allows for analyzing rare treatments such as CAR-T. The NIS was developed for the Healthcare Cost and Utilization Project (HCUP; www.hcup-us.ahrq.gov) and the Agency for Healthcare Research and Quality (AHRQ) to estimate inpatient utilization, access, cost quality and in-hospital outcomes [6]. The publicly available, all-payer, inpatient database contains de-identified data from more than seven million hospital stays annually [6]. For this analysis of the NIS, we followed the Strengthening the Reporting of Observational Studies in Epidemiology (STROBE) statement for reporting observational studies [7]. In accordance with the Declaration of Helsinki, our local study NIS research committee deemed local Institutional Review Board (IRB) review was not necessary given the de-identified nature of the data.

We first identified all NIS hospitalizations containing a cancer diagnosis (including principal as well as secondary diagnoses) with a Food and Drug Administration (FDA) approved indication for CAR-T [8, 9]. Cancers eligible for inclusion were selected using the International Classification of Diseases, Tenth Revision, Clinical Modification (ICD- 10-CM) and consisted of DLCBL, B-cell precursor acute lymphoblastic leukemia, follicular lymphoma, mantle cell lymphoma, and multiple myeloma (Supplementary Table 1) [9]. Among these eligible hospitalizations, ICD- 10 procedure codes identified patients undergoing CAR-T (Supplementary Table 1). Eligible patients were further divided by the presence or absence of AF or atrial flutter (principal or secondary diagnosis) at any time by ICD- 10-CM codes I48.0, I48.1, I48.2, I48.3, I48.4, I48.91, and I48.92. Notably, the NIS does not include patients receiving CAR-T in clinical trials or outpatient infusion clinics, and these patients were naturally excluded.

We collected patient demographics for each hospital stay including age, sex, race, median household income by zip code, hospital region, hospital bed number and insurance status. Hospital bed number was defined as large (hospitals with > 299 beds), medium (hospitals with 100–299 beds), small (hospitals with < 100 beds). Patient comorbidity status was assessed using the Charlson Comorbidity Index (CCI), a point-based system with values of 0, 1, 2 or ≥ 3 representing no, mild, moderate, and severe comorbid burden, respectively [10]. The primary outcome for the study was in-hospital mortality. Secondary outcomes were length of hospital stay as well as the occurrence of in-hospital gastrointestinal bleeding, pulmonary edema, and/or acute heart failure. We additionally reported on the association of atrial fibrillation with respiratory failure and sepsis as these conditions can often coexist. These outcomes and diagnoses of interest were identified using ICD- 10-CM codes found in any principal or secondary diagnosis field [6]. The ICD- 10-CM diagnosis and ICD- 10 procedure codes used for the study are shown in Supplementary Tables 1 and 2.

We evaluated differences between CAR-T patients with and without AF using Chi-squared or Fisher's exact tests for categorical variables (e.g., sex, in-hospital mortality, race, comorbidity index categories), and Student’s t-test for the length of stay (LOS). Univariable logistic regression models assessed the association by odds ratio with AF and outcomes (i.e., in-hospital mortality) as well as other baseline socio-demographics and comorbidities that have been associated with in-hospital mortality (e.g., age, sex obtained from medical records as male or female assigned at birth, race, comorbidity index, hospital bed number, hospital region, hospital teaching status, and household income national quartiles). Variables with a *p*-value of ≤ 0.20 in the unadjusted weighted univariate regressions were subsequently included in a multivariable logistic regression model [11]. Age and sex were included into the multivariable regression model independent of the univariable regression model due to the significance of these variables in our primary and secondary outcomes of interest [11]. Additional multivariable logistic regression models tested the independent association of atrial fibrillation with secondary outcomes using the same covariates. For length of stay (LOS), we used negative binomial regression to assess association (by beta coefficient) of AF with LOS, after an evaluation of the dispersion of LOS variable in the histogram showed over-dispersed data for the LOS in days.

We defined survey parameters (weight, strata, and stratum) to account for the NIS complex survey sampling methods [6]. This study addressed the potential for misclassification bias by using ICD- 10 codes validated in prior studies as well as codes used in prior published papers [12–16]. The multiple imputation method was planned for imputed missing data if more than ten percent of variables were missing [17].

Since differences among cancer types could introduce variation into the study findings, we performed a sensitivity analysis in the subgroup of patients with diffuse large B-cell lymphoma which constituted 70% of the patients receiving CAR-T. Since sepsis and respiratory failure could be confounding factors that contribute to the development of atrial fibrillation as well as the measured outcomes, we performed an additional sensitivity analysis excluding patients with these conditions.

For reference, descriptive statistics were employed to report cardiovascular and bleeding diagnoses associated with all hospitalizations, with and without CAR-T, among the patients with cancers with an FDA-approved indication for CAR-T. Only descriptive statistics are reported, given potential variations in hospitalization indications. (All CAR-T therapy admissions would be expected to be specifically for CAR-T therapy, while other hospitalizations may have been for indications unrelated to cancer treatment such as sepsis.)

In this analysis, we stressed the precision of the study estimates, focusing on 95% confidence intervals. *P*-values are presented to aid interpretation and are non-adjusted. All analyses were performed with STATA 17.0 (Stata-Corp LP, College Station, Texas).

## Results

**Fig. 1:**
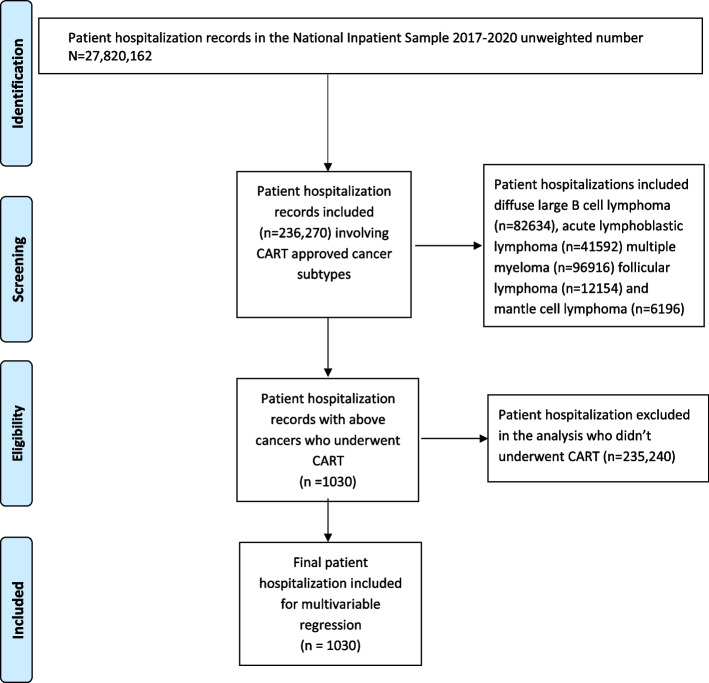
Flow diagram of study population selection with information on inclusion observations. Abbreviations: CAR-T: Chimeric Antigen Receptor T-cell

**Fig. 2:**
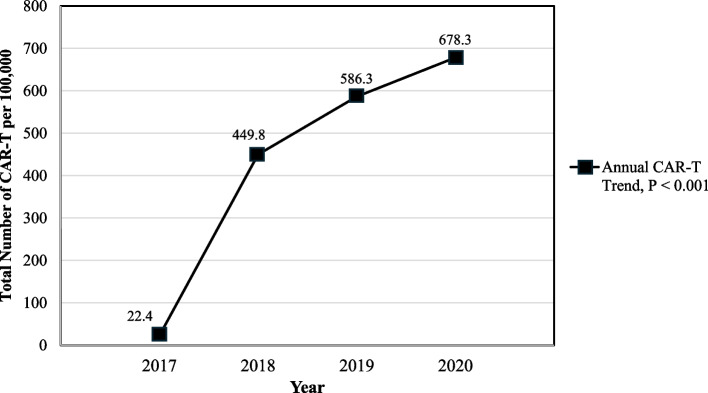
Trend of Chimeric Antigen Receptor T-cell Therapy (CAR-T) hospitalizations

Among the 236,270 hospitalizations involving CAR-T approved cancer subtypes (DLBCL, multiple myeloma, follicular lymphoma, mantle cell lymphoma, and acute lymphoblastic leukemia), 1,030 (0.44%) hospitalizations involved administration of CAR-T between January 1, 2017, and December 31, 2020 (Fig. 1, Supplementary Table 3). These 1,030 unweighted hospitalizations represented approximately 4,670 records after applying weights for national estimates. CAR-T hospitalizations steadily rose from 2017–2020 (Fig. 2, Supplementary Table 3). DLBCL accounted for 70% of patients receiving CAR-T (Supplementary Table 4).

Among the 1,030 unweighted hospitalizations, 43 records were missing race, 45 records were missing income quartiles, and 8 records were missing insurance status. (Multiple imputations were not indicated per the statistical plan, as the total records with missing variables were less than ten percent.)

Of the 1,030 patients receiving CAR-T (mean age 55.6 ± 18.1 years), 97 (9.4%) had an associated diagnosis of AF during their hospitalization. Compared to CAR-T patients without AF, those with AF were found to be older (mean age 68 vs. 54 years, *p* < 0.001), more likely to be male (42% vs. 22%, *p* < 0.001), more likely to be Caucasian (84% vs. 70%, *p* < 0.001), have Medicare (57% vs. 30%, *p* < 0.001), and have a higher prevalence of 3 or more comorbidities by the Charlson index (59% vs. 38%, *p* < 0.001) (Table 1). CAR-T patients with AF were also more likely to have a diagnosis of hypertension (56.7% vs 36.6%, *p* < 0.001), chronic heart failure (24.7% vs 7%, *p* < 0.001), and coronary artery disease (18.6% vs 5%, *p* < 0.001) (Table 1).

**Table 1:** Baseline socio-demographics and comorbidity Index of CAR-T patients with and without atrial fibrillation

**Baseline Demographics and Comorbidities**	**Variables **	**Total Study***n***= 1030**	**CAR-T with AF** *n***= 97 (9.4%)**	**CAR-T without AF** *n***= 933 (90.6%)**	***P*-value**
**Age (years) ±SD**	**-**	55.6 ± 18.1	68.2 ± 9.4	54.3 ± 18.3	**< 0.001**
**Gender,** *n* **(%)**	Male	613 (59.5)	75 (78)	538 (57.6)	**< 0.001**
	Female	417 (40.5)	21 (22)	396 (42.4)	
**Race,** *n* **(%)**	Caucasian	704 (71)	79 (84)	625 (70)	**< 0.001**
	Black	63 (6.4)	-	-	
	Hispanic	125 (12.7)	-	-	
	Asian	40 (4)	-	-	
**Insurance,** *n ***(%)**	Medicare	335 (32.6)	55 (57)	280 (30)	**< 0.001**
	Medicaid	105 (10.2)	-	-	
	Private	524 (51.0)	33 (34)	491 (52.7)	
	Self-pay	27 (2.6)	-	-	
**Region,** *n ***(%)**	Northeast	283 (27.5)	24 (25)	259 (27.7)	0.255
	Midwest	245 (23.8)	22 (23)	223 (24)	
	South	268 (26.0)	33 (34)	235 (25)	
	West	234 (22.7)	17 (17.7)	217 (23)	
**Setting/location,** *n* **(%)**	Rural	-	-	-	1.00
	Urban non-teaching	-	-	-	
	Urban teaching	1023 (99.3)	96 (100)	927 (99.3)	
**Bed size,** *n ***(%)**	Small	129 (12.5)	14 (14.6)	115 (12.3)	0.055
	Medium	143 (13.9)	-	-	
	Large	758 (73.6)	76 (79)	682 (73)	
**Annual income (US $ per year),** *n* **(%)**	1–45,999	175 (17.8)	16 (17)	159 (18)	0.229
	46 K–58,999	205 (20.8)	18 (19)	187 (21)	
	59 K- 78,999	276 (28)	20 (21)	256 (28.7)	
	79 K or more	329 (33.4)	40 (42.6)	289 (32)	
**Charlson comorbidity index,** *n ***(%)**	0	-	-	-	**< 0.001**
	1	-	-	-	
	2	614 (59.6)	38 (40)	576 (61.7)	
	3 or higher	413 (40.1)	57 (59)	356 (38)	
**Cardiovascular Risk Factors and Disease**	Hypertension	396 (38.4)	55 (56.7)	341 (36.6)	**< 0.001**
	Obesity	70 (6.8)	-	-	0.671
	CAD	65 (6.3)	18 (18.6)	47 (5)	**< 0.001**
	Chronic Heart Failure	89 (8.6)	24 (24.7)	65 (7)	**< 0.001**

Comparison of baseline socio-demographics, Charlson Comorbidity Index, and cardiovascular risk factors and disease in patients admitted for CAR-T with and without atrial fibrillation. Data reflects all hospitalizations from the National Inpatient Sample between 2017 - 2020 with an FDA approved indication for CAR-T. Cancer types included are diffuse large B cell lymphoma, B-cell precursor acute lymphoblastic leukemia, follicular lymphoma, mantle cell lymphoma, and multiple myeloma. Frequencies less than 11 are marked as “-” due to Healthcare Cost and Utilization Project (HCUP) guidelines. The Student t-test assessed differences in continuous variables while Chi-squared assessed differences in categorical variables

*Abbreviations:* *AF* Atrial fibrillation, *CAD* Coronary Artery Disease, *CCI* Charlson Comorbidity Index, *N/A* Non-applicable. As per National inpatient sample we cannot report numbers less than or equal to 11 so in such cases N/A has been used. *CAR-T* Chimeric Receptor Antigen T-cell therapy, *FDA* Food and Drug Administration, *US* United States, *SD* Standard Deviation

In-hospital cardiovascular, bleeding, and infectious diagnoses are listed in Table 2. Compared to CAR-T without AF, CAR-T with AF was associated with a higher prevalence of hypotension (35% vs 28%, *p* = 0.157), supraventricular arrhythmia (*p* = 0.001), acute heart failure (*p* < 0.001), pulmonary edema (*p* = 0.009), acute myocardial infarction (*p* = 0.048), gastrointestinal bleed (*p* = 0.028), disseminated intravascular coagulation (*p* = 0.006), and sepsis (15.5% vs 8.5%, *p* = 0.039). In the tables, frequencies less than 11 are not specifically reported as per the data reporting and publishing policy of HCUP [6, 12].

**Table 2:** In-hospital cardiovascular, bleeding and infectious diagnoses associated with CAR-T with and without AF

		**Total Study***n***= 1030**	**CAR-T with AF** *n***= 97 (9.4%)**	**CAR-T without AF** *n***= 933 (90.6%)**	***P*-value**
**Pericardial Disease**	Pericardial disease including pericarditis	15 (1.5)	-	-	0.645
**Arrhythmias**	Supraventricular tachycardia	35 (3.4)	⬆	-	**0.001**
	Ventricular arrhythmias	44 (4.3)	⬆	-	0.059
**Acute Heart failure**	Acute Heart Failure	12 (1.2)	⬆	-	**< 0.001**
	Pulmonary Edema	33 (3.2)	⬆	-	**0.009**
**Acute myocardial infarction (AMI)**	AMI	29 (2.8)	⬆	-	**0.048**
**Thromboembolism**	Acute DVT and PE	20 (1.9)	-	-	0.712
**Hypotension and Critical Care**	Hypotension	295 (28.6)	34 (35.1)	261 (27.9)	0.157
	Mechanical ventilation requirement	45 (4.4)	⬆	-	0.063
**Bleeding Complications**	GI Bleed	18 (1.7)	⬆	-	**0.021**
	DIC	38 (3.7)	⬆	-	**0.006**
**Sepsis**		94 (9.1)	15 (15.5)	79 (8.5)	**0.039**

Comparison of inpatient cardiovascular and bleeding diagnoses in patients admitted for CAR-T with and without atrial fibrillation. Data reflects all hospitalizations from the National Inpatient Sample between 2017 - 2020 with an FDA approved indication for CAR-T. Cancer types included are diffuse large B cell lymphoma, B-cell precursor acute lymphoblastic leukemia, follicular lymphoma, mantle cell lymphoma, and multiple myeloma. Supraventricular tachycardia, acute heart failure, pulmonary edema, acute myocardial infarction, gastrointestinal bleeding, and disseminated intravascular coagulation were more common in atrial fibrillation patients. There was a trend towards increased ventricular arrhythmias in patients with AF. Variables with a frequency less than 11 are not specifically annotated due to Healthcare Cost and Utilization Project (HCUP) reporting policy on low numbers. (⬆ represents the cohort with the higher frequency for comparison if a significant difference or strong trend exists. “-” otherwise marks both the cell in question and the corresponding cell to prevent computation of the missing cell count)

*Abbreviations:* *CAR-T* Chimeric Receptor Antigen T-cell Therapy, *DVT* Deep Vein Thrombosis, *DIC* Disseminated Intravascular Coagulation, *FDA* Food and Drug Administration, *GI* Gastro-Intestinal, *AMI* Acute Myocardial Infarction includes (*STEMI* ST Segment Elevation Myocardial Infarction, *N/A* Not Available, *NSTEMI* Non-ST Segment Elevation Myocardial Infarction, and *UA* Unstable angina), *PE* Pulmonary Embolism, *SD* Standard Deviation, *SVT* Supraventricular Tachycardia

There was a total of 39 in-hospital deaths in the overall cohort. Univariable logistic regression models assessed the associations of in-hospital mortality in CAR-T admissions with age, sex, atrial fibrillation, race, hospital region, hospital bed size, household income, year, Charlson comorbidity, and insurance status (Table 3). Atrial fibrillation and Charlson comorbidity index were both independently associated with increased in-hospital mortality. A multivariable model (Table 3) analyzed the independent association of AF with hospitalization after adjusting for age, sex, race, household income, and Charlson comorbidity (included in the model given univariable *p*-values were ≤ 0.20). The adjusted odds of in-hospital mortality (the primary outcome) were more than three times higher in the group of CAR-T recipients with AF compared to the CAR-T group without AF (aOR: 3.87, 95% CI: 1.61, 9.30,* p* = 0.003) (Tables 3 and 4).

**Table 3:** Unadjusted and adjusted odds ratios for in-hospital mortality during CAR-T hospitalization in the NIS 2017 - 2020

**Variables**		**Univariable Logistic Regression**			**Multivariable Logistic Regression**		
		**OR**	**95% CI**	***P-*value**	**aOR**	**95% CI**	***P-*value**
**Age**		0.99	0.97–1.01	0.181	0.98	0.96–1.01	**0.017**
**Atrial fibrillation**		3.12	1.43–6.80	**0.004**	3.87	1.61–9.30	**0.002**
**Female Sex**		0.82	0.42–1.60	0.554	0.99	0.51–1.93	0.975
**Race**	Ref: Caucasian			0.069			**< 0.001**
	African American	2.01	0.66–6.07		1.8	0.63–5.10	
	Hispanic	0.98	0.34–2.85		0.77	0.21–2.85	
	Asian	1.56	0.37–6.60		1.75	0.38–8.07	
**Region**				0.859			-
**Bed size**				0.417			-
**Annual income (US$ per year)**	Ref: 1–45,999			0.071			**< 0.001**
	46 K–58,999	0.36	0.89–1.42		0.37	0.09–1.57	
	59 K- 78,999	0.62	0.26–1.51		0.69	0.28–1.74	
	79 K or more	1.39	0.53–3.62		1.59	0.55–4.67	
**Year**				0.377			-
**Charlson Comorbidity**		1.99	1.10–3.67	**0.028**	1.86	0.92–3.73	0.083
**Insurance**				0.774			-

Univariable and multivariable logistic regression assessed the association of baseline socio-demographics, charlson-comorbidity index and atrial fibrillation with in-hospital mortality. Variables were included in the multivariable model if the unadjusted *p*-value was £ 0.20

*Abbreviations:* *NIS* National Inpatient Sample, *CAR-T* Chimeric Receptor Antigen T-cell Therapy, *LL* lower limit, *UP* upper limit, *N/A* Not application in multivariate regression, *OR* Odds ratio, *USD* United States Dollar

**Table 4:** Adjusted odds ratio (effect) of various in-hospital outcomes in CAR-T AF group compared to CAR-T non-AF group

**In-hospital outcomes**	**Adjusted OR**	**95% CI**	***P*-value**
In-hospital mortality	3.87	1.61–9.30	0.002
Pulmonary edema	3.29	1.34–8.09	0.010
Gastrointestinal bleed	5.46	1.95–15.29	0.001
Acute Heart Failure	10.2	2.15–47.95	0.003
Sepsis	2.12	1.18–3.82	0.012
Respiratory Failure	2.93	1.29–6.68	0.010
	**Beta Coefficient**	**95% CI**	***P*-value**
Length of stay	0.18	0.01–0.36	0.045

Adjusted odds ratios and beta coefficient after multivariable logistic regression and negative binomial regression in primary and secondary outcomes adjusted for age, sex, race, comorbidity, and income. The median length of stay in CAR-T with AF was 23 days compared to 20 days in the CAR-T group without AF

*Abbreviations:* *AF* Atrial Fibrillation, *CAR-T* Chimeric Antigen Receptor T-cell therapy, *CI* Confidence Interval, *OR* Odds Ratio, *LL* Lower limit, *UL* Upper Limit

Similar multivariable regression models were used for the secondary outcomes with the exception of length of stay, which was evaluated with negative binomial regression. The adjusted odds of pulmonary edema were more than three times higher (aOR: 3.29, 95% CI: 1.34, 8.09,* p* = 0.010), adjusted odds of gastrointestinal bleed were more than five times higher (aOR: 5.46, 95% CI: 1.95, 15.29,* p* = 0.001), and adjusted odds of acute heart failure more than ten times higher (aOR: 10.20, 95% CI: 2.15, 47.95,* p* = 0.003) in CAR-T with AF compared to CAR-T without AF (Table 4). Length of stay was longer in CAR-T with AF versus without AF (mean length of stay 20.5 days vs 7.5 days, *p* < 0.001, adjusted beta co-efficient 0.18, 95% CI: 0.01, 0.36, *p* = 0.045) as also shown in Table 4. Again adjusting for the same covariates, multivariable regression models also evaluated the association of atrial fibrillation with respiratory failure (aOR 2.93, 95% CI 1.29–6.68) and sepsis (aOR 2.12, 95% CI 1.18–3.82) (Table 4).

The demographics, cancer types, in-hospital CV and bleeding diagnosis associated with CAR-T vs non-CAR-T in the five FDA-approved cancer types are reported for reference. Only descriptive statistics are used given the potential differences in hospitalization indications in patients not receiving CAR-T. Among the 236,270 hospitalizations with cancer subtypes where CAR-T is approved, only 0.4% of hospitalizations included the administration of CAR-T. The majority of CAR-T admissions were in patients with DLBCL (70.2%) followed by acute lymphoblastic leukemia (12.3%), multiple myeloma (11.6%) and mantle cell lymphoma (2.2%). (Supplementary Table 4).

Compared to patients not receiving CAR-T, patients receiving CAR-T had numerically higher incidence of hypotension (28.6% vs. 8.1%), ventricular tachycardia (3.2% vs. 1.5%), supraventricular tachycardia (3.4% vs. 1.8%), pulmonary edema (3.2% vs. 1.1%), mechanical ventilation requirement (4.4% vs 2.8%) and disseminated intravascular coagulation (3.7% vs. 2.4%) (Supplementary Table 5).

CRS was diagnosed in 60 patients out of 138 in the study cohort (unweighted) after October 2020, when the ICD 10 code for CRS was introduced [16]. Among them, 9 out of 13 patients (69%) developed CRS in the AF group compared to 51 out of 125 patients (41%) who developed CRS in the non-AF group, *p* = 0.08. Tocilizumab administration was captured in the NIS after August 1, 2020, and was delivered to 5 patients (unweighted) during this time period (1 in AF group and 4 in non-AF group).Given the potential impact of cancer type on outcomes, we performed a sensitivity analysis of our AF and CAR-T limited to patients with DLBCL alone, who constituted 70% of CAR-T patients (Supplementary Tables 4 and 6). In a multivariable logistic regression model in the subgroup of DLBCL patients, the results were similar to the entire cohort (Supplementary Table 7). The odds of in-hospital mortality (the primary outcome), when adjusted for age, race, sex, Charlson comorbidity, and income were more than five times higher in CAR-T with AF compared to CAR-T without AF (aOR: 5.84, 95% CI: 2.36, 14.47,* p* < 0.001) (Supplementary Table 6). Similarly, the adjusted odds of gastrointestinal bleeding were more than four times higher (aOR: 4.87, 95% CI: 1.13, 21.04,* p* = 0.034), and the adjusted odds of acute heart failure more than seven times higher (aOR: 7.99, 95% CI: 1.54, 41.40,* p* = 0.013) in CAR-T with AF compared to CAR-T without AF. While there was a similar trend for increased pulmonary edema (aOR: 2.73, 95% CI: 0.79, 9.36,* p* = 0.111) and length of stay (adjusted beta co-efficient 0.15, 95% CI: − 0.06, 0.35, *p* = 0.157) in the CAR-T with AF group compared to CAR-T without AF, the results were not significant (Supplementary Table 6).

To remove potential confounding due to the presence of sepsis (n = 94) and respiratory failure (*n* = 45), we conducted a separate sensitivity analysis in the 913 patients remaining after excluding these conditions from the overall CAR-T cohort. The results of the sensitivity analysis were similar to the primary analysis (Supplementary Table 8). There remained a strong association with AF on mortality (aOR 21.4, 95% CI 3.85–118.85). There were also trends towards more pulmonary edema, acute heart failure, and longer length of stay in line with the main analysis. There were no gastrointestinal bleeding events in this reduced cohort, in either patients with or without AF.

## Discussion

In a large cohort of 1,030 hospitalizations for CAR-T in the NIS, representing 4670 unweighted hospitalizations, AF is common and is independently associated with increased in-hospital mortality, gastrointestinal bleeding, acute heart failure, pulmonary edema, and increased length of stay. While prior studies have commented on the frequency and rates of cardiovascular events after CAR-T in smaller cohorts or the pharmacovigilance database, no studies have assessed the impact of comorbid AF on inpatient mortality and associated events in the CAR-T population [5, 18–20]. Thus, our study sheds light on the impact of AF on CAR-T outcomes and highlights the potential importance of reducing AF burden in the CAR-T population.

Multiple lines of evidence indicate that AF is a predictor of in-hospital mortality in non-CAR-T patients with metastatic cancers, DLBCL, and multiple myeloma [21, 22]. In an analysis of the FDA pharmacovigilance database, Goldman et al. reported that hypotension and AF were the most frequent cardiovascular events reported with CAR-T and were thought to be likely secondary to CRS [5]. Limitations of the pharmacovigilance database, however, prevented computing of incidence or odds ratios. Our study thus helps improve our understanding of the incidence of inpatient AF with CAR-T and its association with a threefold increased risk of in-patient mortality and increased length of stay.

In patients receiving CAR-T, AF can occur as part of cytokine release syndrome (CRS) or independently [4]. CRS classically presents with a combination of high fever, severe hypotension, and hypoxia occurring within a few days of CAR-T infusion. We were not able to fully assess the association of CRS with AF as the ICD- 10 code for CRS was not available until October, 2020, near the end of the study period. After the code’s implementation, there was a trend for a higher rate of CRS in the AF group vs the non-AF group (69% vs 41%; *p* = 0.08). These proportions are similar to a single center study of 213 patients at Memorial Sloan Kettering that showed CRS to be significantly more likely in patients with atrial arrhythmias vs those without following CAR-T (78% vs 43%, *p* < 0.001) [20]. (Atrial arrhythmias were defined as AF, atrial flutter and supraventricular arrhythmia in this study). In the Memorial Sloan Kettering cohort, 11 of the 23 patients (48%) who developed atrial arrhythmias after CAR-T had pre-existing atrial arrhythmias with the remainder being de-novo. Having a pre-existing atrial arrhythmia was the strongest multivariable predictor of experiencing an atrial arrhythmia after CAR-T (OR = 6.80 [2.39–19.6]) [20]. While the MSK study cohort did not show a difference in overall survival in patients with atrial arrhythmias vs those without, the analysis was limited to a landmark analysis beginning 30 days after CAR-T administration. Thus, inpatient mortality was not reported, and the survival analysis only applies to patients alive at 30 days. While further studies will need to continue to investigate the underlying etiologies of AF (CRS-dependent and independent pathways), we are able to show that AF, regardless of the underlying cause, is strongly associated with inpatient mortality and important secondary outcomes.

Pulmonary edema and decreased left ventricular ejection fraction have also been reported in 6% and 10% of CAR-T recipients in the context of high-grade CRS [23, 24]. These previous findings suggest that the higher incidence of adverse events, such as pulmonary edema and acute heart failure of CAR-T patients with AF observed in our study could be related to CRS. Several case reports have also proposed CRS-independent mechanisms linking CAR-T and cardiotoxicity. Indeed, autopsies in at least two patients who received CAR-T uncovered high concentrations of CAR T-cells in the myocardium and pericardial fluid [25]. This suggests that cardiotoxic CAR T-cells may be a possible culprit in CAR-T recipients who develop cardiopulmonary symptoms and arrhythmia. As such, more extensive studies are needed to determine CRS-dependent and -independent mechanisms in CAR-T patients who develop cardiac dysfunction [26].

Patients within the CAR-T group with AF were notably older than their counterparts without AF, (68.2 vs. 54.3-year-old, *p* < 0.001). Age is a well-established independent predictor of mortality and is significantly associated with AF [27]. There is also a strong link between mortality (in both sexes, irrespective of age), bleeding risk, and higher CCI with AF [19, 28, 29]. To our knowledge, we are the first to report data involving these metrics in CAR-T patients.

Hypertension, chronic heart failure and coronary artery disease were prevalent in the CAR-T AF group, and there certainly may be opportunities for improved cardiovascular optimization prior to CAR-T. Certainly, baseline cardiovascular examinations have been recommended in patients at risk for complications during CAR-T [30, 31]. Importantly, to date no CV comorbidity has been identified as a specific contraindication to CAR-T, but optimization of these risk factors should help reduce risk of events during treatment.

Patients with AF were also more likely than CAR-T patients without AF to be diagnosed with acute heart failure, pulmonary edema, acute myocardial infarction, gastrointestinal bleeding, and disseminated intravascular coagulation during their hospitalization. Due to limitations with the NIS, we cannot discern the relative order that these events occurred. However, regardless of the initial driver, the occurrence of atrial fibrillation clearly identifies patients at substantially higher risk of prolonged hospitalization and increased inpatient mortality.

Management of atrial fibrillation can be challenging in cancer patients. Thrombocytopenia can limit use of anticoagulation and thus prevent safe cardioversion, while use of anti-arrhythmic therapies can be further complicated by other QT prolonging medications or drug-drug interactions. The general framework for management of atrial fibrillation in cancer patients currently resembles that for the general population, [32] but further studies are needed to address lingering questions. Given the relatively high rate of atrial fibrillation in CAR-T patients, is premedication with anti-arrhythmic warranted? Does the presence of cancer treatment influence the optimal anti-arrhythmic option? As an example, amiodarone is generally not first line in the general population due to long-term toxicity, but it may be a safer option in an acute setting given a lower risk for torsades de pointes [33]. For now these questions remain unanswered, but they are of increased importance given the significant impact atrial fibrillation has in the cancer population, including those receiving CAR-T. Further studies should also explore the impact of tocilizumab on reducing in-hospital mortality in patients receiving CAR-T therapy with AF.

Due to limitations with the NIS, we could not differentiate whether AF was present on admission or occurred as a complication from the CAR-T. The results thus reflect associations with AF more broadly, and future research should investigate the impact of AF onset and duration. Our study was also limited to inpatient CAR-T administration and did not include patients in outpatient infusion clinics. While there is a trend towards increased outpatient administration of CAR-T, the administration of CAR-T was predominantly inpatient during the study period. Our findings may not be generalizable to the outpatient settings. Data obtained for this study was limited to hospitalizations before December 31, 2020, and only three CAR-T products (brexucabtagene autoleucel, tisagenlecleucel, and axicabtagene ciloleucel) were available within this period. Thus, findings from this study may not reflect outcome profiles from other CAR-T products. Additionally, the NIS classifies readmissions as new admissions which introduces the potential for double-counting patients. However, the impact would be minimal for our analysis as every hospitalization included the administration of CAR-T and readministration of CAR-T would be rare during the study period. During the study period, administration of CAR-T was primarily conducted in urban teaching hospitals with large bed numbers (99%) due to high costs and special equipment requirements [34, 35]. Caucasians represented 71.3% of the study population, limiting our findings'applicability to other patient populations. However, these demographics are consistent with those patients receiving CAR-T. Our data was collected retrospectively, and remaining residual confounders would be expected. The NIS does not have cancer staging information nor cause of death. As deaths were all in-hospital mortality, we would expect them to be more likely treatment related adverse effects, including cardiovascular, rather than cancer progression. The administrative code for CRS was introduced October 2020, and CRS could not be assessed prior to this date. The potential for misclassification bias is also a known concern with the use of administrative (ICD- 10) codes. We did use previously validated codes to minimize bias and maximize comparability. Additionally, there was no systematic surveillance for AF and the incidence of inpatient AF, especially asymptomatic AF, could be underestimated. However, presumably all clinically significant AF would have been recorded. Finally, our study could not analyze outcomes based on cancer stage or grading of incident CRS.

## Conclusions

The presence of AF in patients undergoing CAR-T is associated with heightened risks of in-hospital mortality, acute pulmonary edema, gastrointestinal bleeding, acute heart failure, and prolonged hospitalization. Further research into AF prevention and treatment has the potential to improve patient outcomes.

### Data availability

The National Inpatient Sample database from 2017 to 2020 in this study is available from the Healthcare Cost and Utilization Project (HCUP) website www.hcup-us.ahrq.gov in the purchase database link (https://cdors.ahrq.gov/databases). HCUP is developed for the Agency for Healthcare Research and Quality (AHRQ) to estimate inpatient utilization, access, cost quality and in-hospital outcomes.

## Supplementary Information

Supplementary Material 1

## Data Availability

This retrospective, population-based cohort observational study used database from all hospitalizations in the National inpatient sample (NIS) from January 1, 2017, to December 31, 2020. NIS is the largest inpatient database in the United States. The NIS was developed for the Healthcare Cost and Utilization Project (HCUP) and the database is publicly available at www.hcup-us.ahrq.gov for purchase from the Agency for Healthcare Research and Quality (AHRQ).

## Abbreviations

AF: Atrial Fibrillation
AHRQ: Agency for Healthcare Research and Quality
AMI: Acute Myocardial Infarction
CAR-T: Chimeric Receptor Antigen T-cell Therapy
CAD: Coronary Artery Disease
CCI: Charlson Comorbidity Index
CRS: Cytokine Release Syndrome
DVT: Deep Vein Thrombosis
DIC: Disseminated Intravascular Coagulation
DLBCL: Diffuse Large B-cell Lymphoma
FDA: Food and Drug Administration
GI: Gastro-Intestinal
HCUP: Healthcare Cost and Utilization Project
ICD: International Classification of Disease
STEMI: ST Segment Elevation Myocardial Infarction
N/A: Not Available
NIS: National Inpatient Sample
NHL: Non-Hodgkin Lymphoma
NSTEMI: Non-ST Segment Elevation Myocardial Infarction
OR: Odds Ratio
PE: Pulmonary Embolism
SD: Standard Deviation
SVT: Supra Ventricular Tachycardia
UA: Unstable angina
