# Supplementary material for: Atrial fibrillation is associated with increased in-hospitality mortality during Chimeric Antigen Receptor T-cell therapy hospitalizations: a retrospective cohort study in the United States
Source: Cardiooncology. 2025 Jul 3;11:60. doi: 10.1186/s40959-025-00334-5 (PMC12224371; doi:10.1186/s40959-025-00334-5)
Supplement: Supplementary file 1 — Supplementary Material 1 [file 40959_2025_334_MOESM1_ESM.docx]

**Supplementary Table 1: Classification of Diagnoses and Procedures by Administrative Coding**

| **Diagnosis or Procedure** | **ICD 10-CM Diagnosis or Procedural Codes** |
| --- | --- |
| **Acute Heart Failure** | I50.21, I50.23, I50.31, I50.33, I50.41, I50.43 |
| **Acute Lymphoblastic Leukemia** | C91.0, C91.00, C91.01, C91.02 |
| **Acute Pulmonary Edema** | I26.01, I26.02, I26.09, I26.90, I26.92, I26.99 |
| **Atrial Fibrillation** | I48.0, I48.2, I48.3, I48.4, I48.91, I48.92 |
| **Brain Hemorrhage (Non-traumatic Intracerebral and Sub-arachnoid Hemorrhage)** | I60.00, I60.01, I60.02, I60.10, I60.11, I60.12, I60.2, I60.30, I60.31, I60.32, I60.4, I60.50, I60.51, I60.52, I60.6, I60.7, I60.8, I60.9, I61.0, I61.1, I61.2, I61.3, I61.4, I61.5, I61.6, I61.8, I61.9 |
| **CAR T-cell Therapy** | XW033C3, XW043C3, XW23346, XW24346, XW23376 XW24376 |
| **Cytokine Release Syndrome** | D89.831, D89.832, D89.833, D89.834, D89.835, D89.839 |
| **Diffuse Large B-cell Lymphoma** | C83.30, C83.31, C83.32, C83.33, C83.34, C83.35, C83.36, C83.37, C83.38, C83.39, C88.4 |
| **Follicular Lymphoma** | C82.00, C82.01, C82.02, C82.03, C82.04, C82.05, C82.06, C82.07, C82.08, C82.09, C82.10, C82.11, C82.12, C82.13, C82.14, C82.15, C82.16, C82.17, C82.18, C82.19, C82.20, C82.21, C82.22, C82.23, C82.24, C82.25, C82.26, C82.27, C82.28, C82.29, C82.30, C82.31, C82.32, C82.33, C82.34, C82.35, C82.36, C82.37, C82.38, C82.39, C82.40, C82.41, C82.42, C82.43, C82.44, C82.45, C82.46, C82.47, C82.48, C82.49, C82.50, C82.51, C82.52, C82.53, C82.54, C82.55, C82.56, C82.57, C82.58, C82.59, C82.60, C82.61, C82.62, C82.63, C82.64, C82.65, C82.66, C82.67, C82.68, C82.69, C82.80, C82.81, C82.82, C82.83, C82.84, C82.85, C82.86, C82.87, C82.88, C82.89, C82.90, C82.91, C82.92, C82.93, C82.94, C82.95, C82.96, C82.97, C82.98, C82.99 |
| **Gastro-intestinal Bleeding** | K92.0, K92.1, K92.2 |
| **Mantle Cell Lymphoma** | C83.10, C83.11, C83.12, C83.13, C83.14, C83.15, C83.16, C83.17, C83.18, C83.19 |
| **Multiple Myeloma** | C90.00, C90.01, C90.02, C90.10, C90.11, C90.12, C90.20, C90.21, C90.22, C90.30, C90.31, C90.32 |

Definitions of comorbidities and procedures based on International Classification of Diseases, Tenth Revision, Clinical Modification (ICD-10-CM) diagnosis and procedure codes.

**Supplementary Table 2: Sepsis Diagnoses and Procedure Coding**

| **Type of Sepsis** | **ICD 10-CM Diagnosis or Procedural Codes** |
| --- | --- |
| Salmonella | A021 |
| Septicemic plague | A207 |
| Anthrax | A227 |
| Erysipelothrix | A267 |
| Listerial | A327 |
| Meningococcal infection, unspecified | A399 |
| Streptococcus, group A | A400 |
| Streptococcus, group B | A401 |
| Streptococcus pneumoniae | A403 |
| Other Streptococcal sepsis | A408 |
| Streptococcal sepsis, unspecified | A409 |
| Methicillin susceptible Staphylococcus aureus | A4101 |
| Methicillin resistant Staphylococcus aureus | A4102 |
| Other Staphlyococcal sepsis | A411 |
| Staphlyococcal sepsis, unspecified | A412 |
| Hemophilus influenzae | A413 |
| Anaerobic | A414 |
| Gram-negative sepsis, unspecified | A4150 |
| Escherichia coli | A4151 |
| Pseudomonas | A4152 |
| Serratia | A4153 |
| Other Gram-negative sepsis | A4159 |
| Enterococcus | A4181 |
| Other Specified Organism | A4189 |
| Unspecified Organism | A419 |
| Actinomycotic | A427 |
| Gonococcal | A5486 |
| Candidal | B377 |
| Severe sepsis without septic shock | R6520 |
| Severe sepsis with septic shock | R6521 |

**Supplementary Table 3: Trend of CAR-T from 2017 to 2020**

| **Year** | **Total number of**  **CAR-T** | **Total hospitalizations with CAR-T approved cancer** | **Total number of CAR-T per 100,000 hospitalizations** |
| --- | --- | --- | --- |
| **2017** | 13 | 57920 | 22.4 |
| **2018** | 267 | 59358 | 449.8 |
| **2019** | 364 | 62088 | 586.3 |
| **2020** | 386 | 56904 | 678.3 |

CAR-T procedures (unweighted) over time in the National Inpatient Sample. The 1,030 total unweighted CAR-T hospitalizations represented approximately 4,670 records after applying weights for national estimates.

Abbreviations:

CAR-T: Chimeric Antigen Receptor T-cell therapy

**Supplementary Table 4: Baseline characteristics of the CAR-T cohort compared to cancer patients without CAR-T in the FDA Approved Cancer Population.**

| **Categories** | **Variables** | **Total  n=236,270(%)** | **CAR-T n=1030 (0.44%)** | **Non-CAR-T n=235,240  (99.56%)** |  |
| --- | --- | --- | --- | --- | --- |
| **Cancer Type** | | Diffuse Large B-Cell Lymphoma | 82634 (35) | 723 (70.2) | 81911 (35.1) |
|  |  | Acute Lymphoblastic Leukemia | 41592 (17.6) | 127 (12.3) | 41465 (17.6) |
|  |  | Multiple Myeloma | 96196 (40.7) | 119 (11.6) | 96077 (40.8) |
|  |  | Follicular Lymphoma | 12154 (5.1) | 66 (6.4) | 12088 (5.1) |
|  |  | Mantle Cell Lymphoma | 6195 (2.6) | 23 (2.2) | 6172 (2.6) |
| **Age (years) ± SD** |  | 60.5±21.9 | 55.6±18.1 | 60.5±21.9 |  |
| **Gender** | Male | 133623 (56.6) | 613 (59.5) | 133010 (56.5) |  |
|  | Female | 102642 (43.4) | 417 (40.5) | 102225 (43.5) |  |
| **Race** | Caucasian | 151648 (66) | 704 (71.3) | 150944 (66) |  |
|  | African American | 32286 (14) | 63 (6.4) | 32223 (14.1) |  |
|  | Hispanic | 28803 (12.5) | 125 (12.7) | 28678 (12.5) |  |
|  | Asian | 7618 (3.3) | 40 (4) | 7578 (3.3) |  |
| **Insurance** | Medicare | 126658 (53.7) | 335 (32.6) | 126323 (53.8) |  |
|  | Medicaid | 29664 (12.6) | 105 (10.2) | 29559 (12.6) |  |
|  | Private | 68346 (29) | 524 (51) | 67822 (28.9) |  |
|  | Self-paying | 4548 (2.0) | 27 (2.6) | 4521 (2.0) |  |
| **Region** | Northeast | 47652 (20.2) | 283 (27.5) | 47369 (20.1) |  |
|  | Midwest | 54119 (23) | 245 (23.8) | 53874 (22.9) |  |
|  | South | 85912 (36.4) | 268 (26.0) | 85644 (36.4) |  |
|  | West | 48587 (20.6) | 234 (22.7) | 48353 (20.6) |  |
| **Setting/location** | Rural | 10529 (4.5) | NA | 10529 (4.5) |  |
|  | Urban non-teaching | 29003 (12.3) | NA | 28996 (12.3) |  |
|  | Urban teaching | 196738 (83.3) | 1023 (99.3) | 195715 (83.2) |  |
| **Bed Size** | Small | 37476 (15.9) | 129 (12.5) | 37347 (15.9) |  |
|  | Medium | 55512 (23.5) | 143 (13.9) | 55369 (23.5) |  |
|  | Large | 143282 (60.6) | 758 (73.6) | 142524 (60.6) |  |
| **Annual income  (US$ per year)** | | 1-45,999 | 57524 (24.8) | 175 (17.8) | 57349 (24.8) |
|  |  | 46K–58,999 | 57832 (24.9) | 205 (20.8) | 57627 (24.9) |
|  |  | 59K-78,999 | 59035 (25.4) | 276 (28) | 58759 (25.4) |
|  |  | 79K or more | 58059 (25) | 329 (33.4) | 57730 (25) |
| **Charlson Comorbidity  Index** | 0 | N/A | N/A | N/A |  |
|  | 1 | N/A | N/A | N/A |  |
|  | 2 | 87960 (37.2) | 614 (59.6) | 87346 (37.1) |  |
|  | 3 or higher | 148297 (62.8) | 413 (40.1) | 147884 (62.9) |  |

Baseline characteristics between CAR-T hospitalizations and non-CAR-T hospitalizations in the FDA Approved Cancer Population. Data reflects all hospitalizations from the National Inpatient Sample (NIS) from 2017-2020 with an FDA approved indication for CAR-T. Diagnoses extracted from principle or secondary diagnoses. N/A: Not applicable based on the Healthcare Cost and Utilization Project (HCUP) guidelines to not to report any number less than 11.

Abbreviations**:** CAR-T: Chimeric Antigen Receptor T-cell therapy SD: Standard Deviation

**Supplementary Table 5. In-hospital Outcomes and Cardiovascular and Bleeding Diagnoses Associated with CAR-T in the FDA Approved Cancer Population**

| **Category** | | **Diagnoses** | **Total  n=236,270(%)** | **CAR-T n=1030 (0.4%)** | **Non-CAR-T n=235,240  (99.6%)** |
| --- | --- | --- | --- | --- | --- |
|  |  |  |  |  |  |
| **In-hospital mortality** |  | 10401 (4.4) | 39 (3.8) | 10362 (4.4) |  |
| **Length of Stay (days)±SD** |  | 7.5±9.3 | 20.5±17.7 | 7.4±9.2 |  |
| **Cardiovascular Risk Factors** | | Hypertension | 133570 (56.5) | 396 (38.4) | 133174 (56.6) |
|  |  | Obesity | 24044 (10.2) | 70 (6.8) | 23974 (10.2) |
| **Cardiovascular Disease** | | Coronary artery disease | 35984 (15.2) | 65 (6.3) | 35919 (15.3) |
|  |  | Pericardial disease including pericarditis | 3488 (1.5) | 15 (1.5) | 3473 (1.5) |
| **Arrhythmias** | | Atrial Fibrillation | 37630 (15.9) | 96 (9.3) | 37534 (16.0) |
|  |  | Supraventricular Tachycardia | 4197(1.8) | 35 (3.4) | 4162 (1.8) |
|  |  | Ventricular Tachycardia | 3481 (1.5) | 33 (3.2) | 3448 (1.5) |
|  |  | Ventricular Fibrillation | 391 (0.2) | N/A | N/A |
| **Heart Failure** | | Acute Heart Failure | 14783 (6.3) | 12(1.2) | 14771(6.3) |
|  |  | Chronic Heart Failure | 48551 (20.6) | 89 (8.6) | 48462 (20.6) |
|  |  | Takotsubo Cardiomyopathy | 305 (0.13) | N/A | N/A |
|  |  | Pulmonary edema | 2688 (1.1) | 33 (3.2) | 2655 (1.1) |

| **Myocardial Infarction** | NSTEMI | 3121 (1.3) | N/A | N/A |
| --- | --- | --- | --- | --- |
|  | STEMI | 470 (0.2) | N/A | N/A |
| **Thromboembolism** | Acute DVT or PE | 4160 (1.8) | 20 (1.9) | 4140 (1.8) |
|  | Splenic Infarct | 440 (0.2) | N/A | N/A |
|  | Arterial Thrombosis | 270 (0.1) | N/A | N/A |
| **Hypotension and Critical Care** | Hypotension | 19315 (8.2) | 295 (28.6) | 19020 (8.1) |
|  | Mechanical ventilation requirement | 6642(2.8) | 45 (4.4) | 6597 (2.8) |
| **Bleeding Complications** | GI Bleed | 4597 (1.95) | 18(1.8) | 4579 (2) |
|  | Brain Hemorrhage | 715 (0.3) | N/A | N/A |
|  | DIC | 5557(2.4%) | 38(3.7) | 5519(2.4) |

In hospital mortality, length of stay, and cardiovascular and bleeding diagnoses in hospitalizations with and without CAR-T administration among the FDA Approved Cancer Population. Data reflects all hospitalizations from the National Inpatient Sample (NIS) from 2017-2020 with an FDA approved indication for CAR-T. Diagnoses extracted from principle or secondary diagnoses. N/A: Not applicable based on the Healthcare Cost and Utilization Project (HCUP) guidelines to not to report any number less than 11.

Abbreviations: CAR-T: Chimeric Antigen Receptor T-cell therapy, DIC: Disseminated Intravascular Coagulation, FDA: Food and Drug Administration, STEMI: ST Segment Elevation Myocardial Infarction, NSTEMI: Non-ST Segment Elevation Myocardial Infarction, SD: Standard Deviation, DVT: Deep Vein Thrombosis, PE: Pulmonary Embolism, SVT: Supra Ventricular Tachycardia, DIC: Disseminated Intravascular Coagulation, GI: Gastro-Intestinal

**Supplementary Table 6. Baseline characteristics and cardiovascular association of CAR-T in Diffuse Large B-Cell Lymphoma population from NIS 2017-2020**

| **Categories** | **Variable** | **Total n=82634 (%)** | **CAR-T  n=723** | **Non-CAR-T  n=81911** |
| --- | --- | --- | --- | --- |
| **Age (years) ±SD** | - | 64.5±16.2 | 60.1±12.9 | 64.5±16.2 |
| **In-Hospital mortality** | - | 3950 (4.8) | 28 (3.9) | 3922 (4.8) |
| **Length of Stay in days** | - | 7.3±8.7 | 19.6±16.2 | 7.2±8.5 |
| **Gender** | Male | 46789 (56.6) | 420 (58) | 46369 (57) |
|  | Female | 35843 (43.4) | 303 (42) | 35540 (43) |
| **Bleeding Complications** | GI Bleed | 1694 (2.1) | 12 (1.7) | 1682 (2) |
|  | Brain Hemorrhage | 286 (0.4) | N/A | 282 (0.3) |
|  | DIC | 2010 (2.4) | 21 (2.9) | 1989 (2.4) |
| **Heart Failure** | Takotsubo Cardiomyopathy | 119 (0.1) | N/A | 115 (0.1) |
|  | Pulmonary edema | 794 (1.0) | 19 (2.4) | 775 (1.0) |
|  | Acute Heart Failure | 3951 (4.8) | N/A | 3942 (4.8) |
|  | Heart Failure | 14494 (17.5) | 70 (9.7) | 14424 (17.6) |
| **Pericardial Disease** | Pericardial disease | 1407 (1.7) | N/A | 1397 (1.7) |
|  | Acute Pericarditis | 54 (0.1) | N/A | 54 (0.1) |
| **Arrhythmia** | Atrial Fibrillation | 12649 (15.3) | 75 (10.4) | 12574 (15.4) |
|  | SVT | 1555 (1.9) | 30 (4) | 1525 (1.9) |
|  | Ventricular Tachycardia | 1216 (1.5) | 27 (3.7) | 1189 (1.5) |
|  | Ventricular Fibrillation | 128 (0.2) | N/A | 128 (0.2) |

Subgroup analysis of DLBCL (largest group of cancer patients receiving CAR-T) including baseline characteristics and in-hospital outcomes which is shown in a combined table supplementary table 6

Abbreviations: NIS: National Inpatient Sample, CAR-T: Chimeric Receptor Antigen T-cell Therapy, LL: lower limit, UP: upper limit, STEMI: ST Segment Elevation Myocardial Infarction, NSTEMI: Non-ST Segment Elevation Myocardial Infarction, SD: Standard Deviation, DVT: Deep Vein Thrombosis, PE: Pulmonary Embolism, CCI: Charlson Comorbidity Index, SVT: Supra Ventricular Tachycardia, DIC: Disseminated Intravascular Coagulation, GI: Gastro-Intestinal

**Supplementary Table 7:** **The Adjusted odds ratio (effect) of various in-hospital outcomes in CAR-T AF group compared to CAR-T non-AF group in subgroup of Diffuse Large B cell lymphoma patients.**

| **In-hospital Outcomes** | **Adjusted OR** | **LL–UL 95% CI** | ***P*-value** |
| --- | --- | --- | --- |
| In-hospital Mortality | 5.84 | 2.36–14.47 | <0.001 |
| Pulmonary Edema | 2.73 | 0.79-9.36 | 0.111 |
| Gastrointestinal Bleed | 4.87 | 1.13–21.04 | 0.034 |
| Acute Heart Failure | 7.99 | 1.54–41.40 | 0.013 |
| Length of Stay (beta coefficient) | 0.15 | -0.06–0.35 | 0.157 |

Multivariable logistic regression and negative binomial regression for adjusted odds ratios adjusted for age, insurance, Charlson comorbidity index, and income.

Abbreviations: AF: Atrial Fibrillation, CAR-T: Chimeric Antigen Receptor T-cell therapy CI: Confidence Interval

**Supplementary Table 8:** **The Adjusted odds ratio (effect) of various in-hospital outcomes in CAR-T AF group compared to CAR-T non-AF group in subgroup excluding sepsis and respiratory failure**

| **In-hospital Outcomes** | **Adjusted OR** | **LL-UL 95% CI** | ***P*-value** |
| --- | --- | --- | --- |
| In-hospital Mortality | 21.4 | 3.85–118.85 | <0.001 |
| Pulmonary edema | 2.92 | 0.88-9.67 | 0.079 |
| Gastrointestinal Bleed | 1 | NA (omitted) | NA (omitted) |
| Acute Heart Failure | 4.65 | 0.74–29.35 | 0.102 |
| Length of Stay (beta coefficient) | 0.15 | -0.04–0.33 | 0.114 |

Adjusted odds ratios and beta coefficient after multivariable logistic regression and negative binomial regression in primary and secondary outcomes adjusted for age, sex, race, comorbidity, and income.

Abbreviations: AF: Atrial Fibrillation, CAR-T: Chimeric Antigen Receptor T-cell therapy, CI: Confidence Interval, OR: Odds Ratio, LL: Lower limit, UL: Upper Limit NA: Not Available omitted due to insufficient/missing variables.

**Supplementary Table 5. In-hospital Outcomes and Cardiovascular and Bleeding Diagnoses Associated with CAR-T with and without AF in patients with Diffuse Large B-cell Lymphoma**

Or

**Unadjusted and Adjusted Odds Ratios for In-Hospital Mortality During CAR-T Hospitalization in the NIS 2017-2020 in patients with Diffuse Large B-cell Lymphoma**

Subgroup analysis of DLBCL (largest group of cancer patients receiving CAR-T) including baseline characteristics and in-hospital outcomes which is shown in a combined table.

Abbreviations: NIS: National Inpatient Sample, CAR-T: Chimeric Receptor Antigen T-cell Therapy, LL: lower limit, UP: upper limit, STEMI: ST Segment Elevation Myocardial Infarction, NSTEMI: Non-ST Segment Elevation Myocardial Infarction, SD: Standard Deviation, DVT: Deep Vein Thrombosis, PE: Pulmonary Embolism, CCI: Charlson Comorbidity Index, SVT: Supra Ventricular Tachycardia, DIC: Disseminated Intravascular Coagulation, GI: Gastro-Intestinal

**Supplementary Table 6:** **The Adjusted odds ratio (effect) of various in-hospital outcomes in CAR-T AF group compared to CAR-T non-AF group in subgroup of Diffuse Large B cell lymphoma patients.**

| **In-hospital Outcomes** | **Adjusted OR** | **LL–UL 95% CI** | ***P*-value** |
| --- | --- | --- | --- |
| In-hospital Mortality | 5.84 | 2.36–14.47 | <0.001 |
| Pulmonary Edema | 2.73 | 0.79-9.36 | 0.111 |
| Gastrointestinal Bleed | 4.87 | 1.13–21.04 | 0.034 |
| Acute Heart Failure | 7.99 | 1.54–41.40 | 0.013 |
| Length of Stay (beta coefficient) | 0.15 | -0.06–0.35 | 0.157 |

Multivariable logistic regression and negative binomial regression for adjusted odds ratios adjusted for age, insurance, Charlson comorbidity index, and income.

Abbreviations: AF: Atrial Fibrillation, CAR-T: Chimeric Antigen Receptor T-cell therapy CI: Confidence Interval
